# Roentgen Rays Are Quite Complex

**Published:** 1897-04

**Authors:** 


Experimenters are discovering that the Roentgen rays are quite complex. One electrician reports in *Nature* that he has differentiated them so as to be quite sure of three distinct kinds. To one kind wood is transparent and flesh is opaque. To another kind bone is almost as transparent as flesh. The suggestion has been made that the difference is due to frequency rather than quality. Another experimenter has succeeded in taking photographs through a plate of iron eight inches thick.

